# Supplementary material for: A Likelihood Approach for Real-Time Calibration of Stochastic Compartmental Epidemic Models
Source: PLoS Comput Biol. 2017 Jan 17;13(1):e1005257. doi: 10.1371/journal.pcbi.1005257 (PMC5240920; doi:10.1371/journal.pcbi.1005257)
Supplement: S1 File — (TAR.GZ) [file pcbi.1005257.s014.tar.gz › HSPH_Online-SI-Revision/IPoi09/n1-severe/n1-severe_BENCH_table4.pdf]

1	R0	{1.51081, 13.7078}	{2.24814, 19.0065}	{{2.20181, 17.7758}, {2.27287, 19.3849}}	{48.8039, 38.6545}	inf	60.	106.646	{60., 170.}	80.4615	0.230198	newly	28.	37.0923	{18., 59.}	51.7033	0.185795	Reff	0.861162	0.561944	{0.355073, 0.834534}	41.5831	ar	656.	853.292	{810., 887.}	30.075
2	R0	{1.73182, 11.3873}	{2.42869, 19.3787}	{{2.41419, 19.1373}, {2.45321, 19.4475}}	{40.2392, 70.178}	inf	73.	102.438	{44., 178.}	59.6172	0.201246	newly	43.	34.	{13., 62.}	42.5932	0.244428	Reff	0.82781	0.491046	{0.322133, 0.784744}	46.4812	ar	653.	879.37	{859., 897.}	34.6661
3	R0	{1.50449, 10.3474}	{2.27015, 19.077}	{{2.23915, 18.4504}, {2.29738, 19.3849}}	{50.8915, 84.3656}	inf	61.	133.721	{85., 189.}	122.107	0.349264	newly	34.	45.3971	{26., 64.}	44.6799	0.172371	Reff	0.825965	0.753826	{0.42961, 1.34626}	38.9132	ar	651.	842.309	{816., 883.}	32.2061
4	R0	{1.63493, 9.1955}	{2.81418, 19.8495}	{{2.7811, 19.6712}, {2.81635, 19.898}}	{72.1284, 115.861}	inf	51.	184.348	{122., 250.}	261.468	0.54189	newly	40.	60.3939	{37., 96.}	56.8939	0.185299	Reff	0.734084	0.56414	{0.321064, 0.843001}	45.2368	ar	663.	924.5	{907., 942.}	39.4419
5	R0	{1.53822, 8.05553}	{2.70845, 19.4959}	{{2.46976, 17.5057}, {2.79269, 19.984}}	{76.077, 142.018}	inf	25.	159.889	{98., 227.}	539.556	0.78391	newly	19.	52.1528	{31., 75.}	174.488	0.41136	Reff	0.772186	0.453957	{0.276002, 0.606014}	42.926	ar	621.	916.028	{891., 936.}	47.5085
6	R0	{1.69374, 17.0793}	{2.16221, 19.7244}	{{2.11476, 19.2116}, {2.1968, 19.9579}}	{27.6591, 15.4869}	inf	95.	133.	{85., 179.}	46.0526	0.171857	newly	35.	45.3036	{28., 63.}	40.2551	0.164158	Reff	0.98745	0.765561	{0.448329, 1.21263}	35.5066	ar	662.	833.75	{794., 872.}	25.9441
7	R0	{1.42179, 11.8031}	{2.28669, 18.9012}	{{2.22731, 17.6755}, {2.39393, 19.6625}}	{60.8317, 60.1373}	inf	48.	90.7164	{53., 148.}	94.2786	0.264028	newly	29.	30.2836	{14., 51.}	36.5414	0.161097	Reff	0.796202	0.491571	{0.346954, 0.667175}	40.9605	ar	517.	857.254	{831., 888.}	65.8131
8	R0	{1.46683, 6.95875}	{2.90402, 18.9176}	{{2.8568, 18.4249}, {2.97157, 19.9817}}	{97.9791, 171.854}	inf	30.	158.529	{97., 220.}	428.429	0.701385	newly	30.	53.	{31., 74.}	79.619	0.236385	Reff	0.545661	0.40542	{0.243669, 0.558558}	34.5751	ar	653.	930.186	{913., 945.}	42.448
9	R0	{1.34525, 11.505}	{2.10308, 18.4888}	{{1.98261, 16.748}, {2.1968, 19.9579}}	{56.3339, 60.7022}	inf	49.	113.763	{59., 171.}	134.313	0.34683	newly	38.	40.4915	{21., 62.}	33.0508	0.140595	Reff	0.881139	0.681058	{0.442183, 1.25324}	33.2805	ar	532.	822.542	{772., 860.}	54.6132
10	R0	{1.77958, 14.372}	{2.42149, 19.4437}	{{2.42205, 19.4475}, {2.42205, 19.4475}}	{36.0709, 35.2886}	inf	58.	125.	{61., 198.}	116.954	0.29844	newly	28.	41.9333	{17., 66.}	65.9524	0.209722	Reff	0.726069	0.591718	{0.337196, 0.88647}	41.4872	ar	671.	878.933	{845., 903.}	30.9886
11	R0	{1.60334, 19.9548}	{2.00254, 19.3741}	{{1.98971, 19.0665}, {1.99771, 19.6444}}	{24.8982, 2.91014}	inf	78.	100.035	{57., 148.}	40.6208	0.145485	newly	24.	33.193	{17., 53.}	49.7076	0.168535	Reff	0.928334	0.677714	{0.419519, 1.02483}	31.6775	ar	617.	800.825	{759., 845.}	29.7933
12	R0	{1.68623, 7.58585}	{2.87071, 15.2753}	{{2.66419, 14.2056}, {2.96947, 15.963}}	{70.2441, 101.366}	inf	21.	98.5135	{47., 169.}	369.112	0.62425	newly	21.	38.0541	{17., 69.}	89.704	0.249417	Reff	0.696413	0.301338	{0.195985, 0.403848}	57.3143	ar	640.	930.095	{909., 952.}	45.3273
13	R0	{1.82689, 18.0022}	{2.27786, 19.2274}	{{2.26161, 19.0355}, {2.29738, 19.3849}}	{24.6852, 6.80573}	inf	97.	114.299	{58., 176.}	38.4213	0.187844	newly	31.	38.5373	{19., 59.}	46.4612	0.203583	Reff	0.811139	0.595447	{0.365283, 0.965707}	40.3012	ar	673.	844.418	{824., 887.}	28.336
14	R0	{1.75911, 9.16883}	{2.89617, 19.0265}	{{2.8568, 18.4249}, {2.97157, 19.9817}}	{64.6383, 107.513}	inf	49.	189.149	{130., 265.}	286.018	0.570985	newly	35.	62.5135	{42., 90.}	79.305	0.235373	Reff	0.809191	0.470553	{0.27711, 0.682241}	46.472	ar	661.	931.189	{913., 949.}	40.8758
15	R0	{1.46168, 11.3859}	{2.41392, 19.3416}	{{2.37462, 19.0531}, {2.42625, 19.5287}}	{65.1471, 69.8729}	inf	42.	72.7324	{27., 139.}	87.9946	0.248325	newly	28.	23.9155	{8., 49.}	49.7988	0.276823	Reff	0.69576	0.400692	{0.288724, 0.542539}	44.3305	ar	556.	880.62	{859., 901.}	58.3848
16	R0	{1.53577, 11.7581}	{2.7544, 18.2507}	{{2.57305, 16.6623}, {2.93978, 19.9817}}	{79.3497, 55.2185}	inf	70.	156.143	{88., 233.}	124.	0.327372	newly	40.	52.7571	{28., 79.}	43.3214	0.151271	Reff	0.7771	0.439254	{0.275439, 0.613388}	46.0299	ar	625.	920.1	{892., 942.}	47.216
17	R0	{1.70698, 9.72924}	{2.86808, 16.8879}	{{2.78517, 15.7559}, {2.96797, 18.4042}}	{68.0208, 73.5787}	inf	45.	129.5	{84., 182.}	187.778	0.435044	newly	22.	46.5	{27., 66.}	112.532	0.303437	Reff	0.626462	0.33544	{0.231512, 0.448163}	46.5325	ar	675.	930.271	{912., 946.}	37.818
18	R0	{1.5973, 14.931}	{1.846, 10.613}	{{1.77826, 9.90957}, {1.91302, 11.3733}}	{15.5697, 28.9199}	inf	48.	83.3636	{30., 132.}	87.0076	0.26839	newly	24.	49.2545	{18., 74.}	114.773	0.315442	Reff	1.13089	0.785046	{0.527857, 1.09042}	36.2201	ar	632.	745.927	{686., 805.}	18.0265
19	R0	{1.87468, 17.004}	{2.26598, 19.3734}	{{2.26161, 19.3211}, {2.27287, 19.3849}}	{20.8731, 13.9345}	inf	76.	92.0714	{45., 146.}	41.3534	0.155496	newly	35.	30.5714	{12., 50.}	37.551	0.1985	Reff	0.783616	0.506103	{0.332457, 0.694314}	44.1149	ar	692.	861.5	{841., 884.}	24.4942
20	R0	{1.84715, 12.531}	{2.28806, 19.2506}	{{2.26161, 19.0355}, {2.30371, 19.3966}}	{23.8697, 53.6236}	inf	47.	90.1538	{45., 147.}	95.6792	0.262305	newly	27.	30.8154	{14., 51.}	44.4444	0.186579	Reff	0.718541	0.479574	{0.319336, 0.728269}	37.5183	ar	661.	859.692	{827., 891.}	30.0594
21	R0	{1.75938, 13.5959}	{2.22521, 12.8652}	{{2.12117, 12.0512}, {2.47412, 14.464}}	{26.477, 8.30458}	inf	73.	66.4394	{31., 115.}	42.3025	0.207265	newly	35.	31.7576	{15., 55.}	39.7403	0.194677	Reff	0.932471	0.440678	{0.326036, 0.569007}	52.7408	ar	620.	846.061	{809., 893.}	36.4614
22	R0	{1.63456, 10.7469}	{2.41972, 19.4458}	{{2.42205, 19.4475}, {2.42205, 19.4475}}	{48.035, 80.943}	inf	44.	107.737	{46., 181.}	145.893	0.342199	newly	29.	35.2982	{13., 66.}	56.8058	0.225271	Reff	0.626036	0.529723	{0.318199, 0.750836}	39.6274	ar	654.	878.93	{845., 905.}	34.3929
23	R0	{1.62702, 19.1392}	{2.32507, 17.7866}	{{2.22731, 16.668}, {2.46477, 19.391}}	{42.9037, 7.59352}	inf	90.	102.164	{49., 177.}	46.8124	0.189535	newly	31.	36.9672	{16., 65.}	52.5648	0.207958	Reff	0.810256	0.536211	{0.317955, 0.902907}	44.277	ar	600.	865.59	{825., 903.}	44.265
24	R0	{1.70466, 12.1202}	{2.32298, 19.003}	{{2.23915, 18.4504}, {2.42625, 19.5287}}	{36.2725, 56.7875}	inf	45.	63.6406	{30., 124.}	64.2014	0.207623	newly	23.	21.7344	{8., 41.}	48.0299	0.236689	Reff	0.758574	0.411343	{0.31056, 0.523961}	46.9754	ar	613.	861.906	{822., 893.}	40.6046
25	R0	{1.61735, 11.3169}	{2.14638, 19.4585}	{{2.12588, 19.2116}, {2.1968, 19.9579}}	{32.7095, 71.9421}	inf	43.	78.7385	{36., 133.}	91.771	0.26394	newly	31.	25.8308	{12., 46.}	37.4194	0.21183	Reff	0.608124	0.567254	{0.346518, 0.790827}	34.2724	ar	681.	834.323	{798., 872.}	22.5144
26	R0	{1.56977, 12.3035}	{2.60291, 18.4626}	{{2.315, 15.4658}, {2.79269, 19.984}}	{65.8147, 50.0594}	inf	36.	99.3333	{51., 150.}	176.543	0.399986	newly	18.	33.3056	{16., 56.}	90.2778	0.252602	Reff	0.846106	0.371924	{0.271265, 0.457907}	56.0428	ar	594.	901.986	{870., 930.}	51.8495
27	R0	{1.46317, 5.8298}	{2.66047, 19.835}	{{2.66225, 19.7855}, {2.66225, 19.9254}}	{81.8292, 240.261}	inf	14.	148.	{84., 227.}	957.143	0.995573	newly	14.	48.1429	{25., 77.}	243.878	0.496553	Reff	0.626237	0.532255	{0.316808, 0.772052}	42.806	ar	586.	910.222	{894., 936.}	55.328
28	R0	{1.70002, 14.9806}	{2.72169, 18.8233}	{{2.47422, 16.1449}, {2.86095, 19.9929}}	{60.0974, 25.703}	inf	101.	161.548	{99., 241.}	64.069	0.202225	newly	48.	53.6935	{31., 76.}	31.8212	0.128354	Reff	0.85511	0.463544	{0.283234, 0.766769}	48.3663	ar	652.	918.435	{897., 940.}	40.8643
29	R0	{1.52729, 16.1014}	{2.26711, 19.353}	{{2.26161, 19.3211}, {2.27287, 19.3849}}	{48.4401, 20.1947}	inf	46.	69.2656	{26., 141.}	72.1128	0.223899	newly	17.	22.75	{8., 47.}	64.1544	0.23033	Reff	0.781972	0.442298	{0.316625, 0.642297}	46.2191	ar	592.	859.172	{836., 885.}	45.1304
30	R0	{1.72795, 13.5635}	{2.42626, 19.4105}	{{2.42205, 19.3966}, {2.42205, 19.4475}}	{40.4124, 43.1085}	inf	61.	89.9857	{34., 161.}	69.1569	0.222969	newly	28.	29.1	{11., 50.}	47.7041	0.218402	Reff	0.661805	0.458456	{0.300334, 0.651531}	38.593	ar	668.	877.986	{850., 897.}	31.435
31	R0	{1.51591, 18.1544}	{2.09165, 17.4073}	{{1.95665, 16.2766}, {2.26107, 19.3211}}	{37.9798, 7.19206}	inf	51.	77.9538	{33., 139.}	65.641	0.207335	newly	16.	27.0462	{12., 47.}	79.6154	0.230171	Reff	0.950476	0.544524	{0.362089, 0.783844}	47.6156	ar	541.	825.	{771., 876.}	52.4954
32	R0	{1.4282, 10.3534}	{1.86387, 13.5623}	{{1.78225, 11.7348}, {2.00043, 15.9062}}	{30.5047, 30.9932}	inf	33.	87.3529	{46., 126.}	164.706	0.386248	newly	26.	41.9608	{23., 62.}	68.3258	0.208704	Reff	1.03687	0.734547	{0.512658, 1.03444}	34.12	ar	521.	753.392	{700., 804.}	44.605
33	R0	{1.56693, 11.3972}	{2.91858, 18.5876}	{{2.8568, 18.0404}, {2.99088, 18.9406}}	{86.261, 63.0889}	inf	84.	170.526	{107., 235.}	103.008	0.290314	newly	50.	58.9474	{37., 85.}	32.0526	0.123524	Reff	0.634607	0.412871	{0.245685, 0.594314}	42.0011	ar	695.	934.237	{917., 949.}	34.4226
34	R0	{1.50675, 19.8823}	{2.11813, 16.3225}	{{2.04464, 14.5648}, {2.22731, 17.695}}	{40.5763, 17.9044}	inf	63.	42.3333	{17., 83.}	48.5392	0.300894	newly	29.	15.8261	{5., 27.}	53.4233	0.376437	Reff	0.797071	0.438332	{0.315768, 0.548981}	45.7109	ar	587.	825.971	{781., 866.}	40.7106
35	R0	{1.72889, 7.76068}	{2.97115, 18.5441}	{{2.96398, 18.1089}, {2.99088, 18.9406}}	{71.8528, 138.95}	inf	43.	172.893	{105., 259.}	302.078	0.581424	newly	34.	58.68	{33., 91.}	74.6275	0.219828	Reff	0.7002	0.412272	{0.246492, 0.649021}	44.7306	ar	650.	935.853	{921., 948.}	43.9774
36	R0	{1.62644, 4.12303}	{2.903, 12.173}	{{2.80569, 11.6897}, {2.96796, 12.4643}}	{78.4881, 195.244}	inf	8.	48.5373	{19., 93.}	506.716	0.698008	newly	9.	22.0597	{9., 41.}	148.756	0.327871	Reff	0.57088	0.248052	{0.173953, 0.322654}	56.5492	ar	652.	930.149	{911., 947.}	42.6609
37	R0	{1.70878, 15.8072}	{2.17296, 19.6218}	{{2.12588, 19.2116}, {2.1968, 19.9579}}	{27.1643, 24.1322}	inf	45.	65.2787	{31., 116.}	61.8944	0.210245	newly	13.	22.3607	{9., 42.}	88.9029	0.293024	Reff	0.772369	0.487208	{0.335889, 0.70737}	42.4064	ar	674.	837.852	{798., 872.}	24.3105
38	R0	{1.58967, 6.80568}	{2.9577, 18.5088}	{{2.86501, 18.0404}, {2.99088, 18.9406}}	{86.0574, 171.961}	inf	25.	128.726	{67., 198.}	414.904	0.673059	newly	22.	43.3288	{22., 70.}	100.809	0.269564	Reff	0.661303	0.331532	{0.221325, 0.454614}	52.2195	ar	610.	935.712	{920., 950.}	53.3955
39	R0	{1.344, 8.19498}	{2.28523, 19.1133}	{{2.23915, 18.4504}, {2.30371, 19.6625}}	{70.0317, 133.232}	inf	37.	118.029	{70., 165.}	221.94	0.518412	newly	28.	40.0294	{20., 58.}	55.8824	0.216228	Reff	0.756672	0.630549	{0.392605, 1.16598}	38.8125	ar	551.	843.426	{814., 887.}	55.869
40	R0	{1.61509, 7.90825}	{2.33121, 10.6635}	{{2.14082, 9.35432}, {2.47628, 11.6528}}	{44.3392, 34.8404}	inf	33.	70.1385	{24., 137.}	124.476	0.313767	newly	23.	38.3692	{13., 72.}	87.8261	0.252654	Reff	0.814005	0.428259	{0.278832, 0.61949}	51.7172	ar	581.	867.154	{809., 903.}	49.252
41	R0	{1.60017, 5.90605}	{2.90668, 19.5971}	{{2.81635, 18.4275}, {2.93978, 19.9817}}	{81.6482, 231.814}	inf	23.	144.316	{79., 243.}	527.46	0.761076	newly	19.	47.	{23., 81.}	147.368	0.348305	Reff	0.611265	0.397323	{0.225751, 0.552679}	45.4317	ar	661.	930.645	{913., 950.}	40.7935
42	R0	{1.64436, 15.1337}	{2.20032, 14.3838}	{{2.10451, 13.1443}, {2.30108, 15.555}}	{33.8099, 6.61931}	inf	68.	80.0189	{43., 139.}	42.7026	0.163742	newly	31.	33.6415	{17., 59.}	40.5356	0.170787	Reff	0.902754	0.479922	{0.335958, 0.608899}	46.838	ar	640.	843.453	{808., 885.}	31.7895
43	R0	{1.48917, 11.917}	{2.11732, 19.518}	{{2.11476, 19.2116}, {2.1968, 19.9579}}	{42.1814, 63.7828}	inf	22.	87.5254	{44., 147.}	300.616	0.571816	newly	12.	28.6102	{12., 49.}	142.09	0.34195	Reff	0.814576	0.56975	{0.388834, 0.78445}	36.9091	ar	551.	824.797	{785., 859.}	49.6909
44	R0	{1.65577, 7.89317}	{2.79833, 19.6286}	{{2.76795, 19.6041}, {2.85956, 19.8999}}	{69.0045, 148.679}	inf	38.	155.675	{97., 235.}	309.672	0.589642	newly	25.	50.2987	{27., 80.}	102.961	0.282622	Reff	0.819606	0.425581	{0.290084, 0.634822}	49.6005	ar	576.	923.558	{907., 941.}	60.34
45	R0	{1.6909, 12.3275}	{2.61191, 19.6728}	{{2.50427, 19.1373}, {2.66225, 19.9254}}	{54.4686, 59.5849}	inf	39.	128.836	{74., 201.}	230.348	0.486715	newly	24.	41.0149	{21., 66.}	77.1144	0.228351	Reff	0.749069	0.4441	{0.309316, 0.671507}	42.3423	ar	675.	901.582	{872., 926.}	33.5677
46	R0	{1.62351, 16.0698}	{2.05934, 18.4825}	{{1.93485, 16.0085}, {2.12588, 19.5406}}	{26.8448, 15.4516}	inf	55.	103.516	{56., 152.}	93.3138	0.269616	newly	26.	37.2097	{17., 57.}	56.6377	0.197648	Reff	1.091	0.673463	{0.435704, 0.985478}	43.3062	ar	591.	807.29	{766., 849.}	33.5973
47	R0	{1.52372, 4.38599}	{2.86686, 14.9262}	{{2.84915, 14.6465}, {2.91192, 15.1048}}	{88.1489, 240.315}	inf	12.	119.015	{55., 213.}	891.793	0.94859	newly	15.	48.8182	{22., 89.}	225.455	0.452469	Reff	0.594251	0.351353	{0.222234, 0.455864}	48.6256	ar	618.	930.788	{914., 944.}	50.6429
48	R0	{1.44306, 11.788}	{2.43245, 18.6754}	{{2.24732, 16.9062}, {2.50427, 19.391}}	{68.5619, 58.4275}	inf	18.	84.2769	{37., 156.}	368.205	0.61811	newly	11.	29.0308	{13., 50.}	164.755	0.364123	Reff	0.793683	0.414219	{0.272166, 0.570583}	49.06	ar	534.	880.723	{849., 913.}	64.9294
49	R0	{1.63624, 11.3893}	{2.88827, 18.4534}	{{2.75274, 17.3228}, {2.97157, 19.3547}}	{76.519, 62.0237}	inf	48.	119.211	{66., 202.}	148.768	0.361615	newly	31.	39.7324	{23., 70.}	41.7083	0.140574	Reff	0.713401	0.3312	{0.228921, 0.478541}	53.7243	ar	643.	930.521	{910., 947.}	44.7156
50	R0	{1.46113, 11.1322}	{2.26193, 18.4588}	{{2.19504, 17.1709}, {2.30371, 19.3966}}	{54.8068, 65.8146}	inf	30.	134.283	{77., 176.}	347.611	0.629257	newly	18.	48.3167	{24., 66.}	168.611	0.402381	Reff	0.936584	0.626349	{0.41577, 0.788248}	36.4844	ar	501.	851.833	{814., 886.}	70.0266
